# New Grain Formation by Constitutional Undercooling Due to Remelting of Segregated Microstructures during Powder Bed Fusion

**DOI:** 10.3390/ma13235517

**Published:** 2020-12-03

**Authors:** Alexander M. Rausch, Martin R. Gotterbarm, Julian Pistor, Matthias Markl, Carolin Körner

**Affiliations:** 1 Chair of Materials Science and Engineering for Metals, Friedrich-Alexander-Universität Erlangen-Nürnberg, Martens Str. 5, 91058 Erlangen, Germany; matthias.markl@fau.de (M.M.); carolin.koerner@fau.de (C.K.); 2 Joint Institute of Advanced Materials and Processes, Friedrich-Alexander-Universität Erlangen-Nürnberg, Dr.-Mack-Str. 81, 90762 Fürth, Germany; martin.gotterbarm@fau.de (M.R.G.); julian.pistor@fau.de (J.P.)

**Keywords:** selective electron beam melting, stray grains, grain structure, equiaxed, nucleation

## Abstract

A microstructure has significant influence on the mechanical properties of parts. For isotropic properties, the formation of equiaxed microstructures by the nucleation of new grains during solidification is necessary. For conventional solidification processes, nucleation is well-understood. Regarding powder bed fusion, the repeated remelting of previous layers can cause nucleation under some conditions that are not explainable with classical theories. Here, we investigate this nucleation mechanism with an unprecedented level of detail. In the first step, we built samples with single crystalline microstructures from Ni-base superalloy IN718 by selective electron beam melting. In the second step, single lines with different parameters were molten on top of these samples. We observed a huge number of new grains by nucleation at the melt-pool border of these single lines. However, new grains can only prevail if the alignment of their crystallographic orientation with respect to the local temperature gradient is superior to that of the base material. The current hypothesis is that nucleation at the melt-pool border happens due to remelting microsegregations from former solidification processes leading to constitutional undercooling directly at the onset of solidification. This study offers the opportunity to understand and exploit this mechanism for different manufacturing processes.

## 1. Introduction

Controlling a microstructure during solidification has been a topic since the beginning of casting. Having columnar, equiaxed, mixed, or single crystalline structures allows for adjusting material and part properties for a given application. However, the margin of controlling microstructures is determined by the manufacturing process and is normally quite narrow. Since the beginning of additive manufacturing, a tool has existed that could exploit different microstructures in one part by adjusting process parameters on the fly. For example, a turbine blade with a customized microstructure in the root and airfoil could be designed. Thus, it would be possible to adjust mechanical properties for local loading conditions [1]. To exploit this freedom in manufacturing, all aspects of microstructural evolution need to be understood.

The usual grain structure for powder-bed-fusion (PBF)-manufactured Ni-based superalloys are columnar grains aligned along the build direction [2]. Therefore, special scan strategies are necessary to gain equiaxed grains for isotropic material properties. Much effort has experimentally and numerically put into understanding microstructural evolution in PBF processes. Common hatching [3], new-point-melting [4,5,6], or ghost-beam [7] scan strategies have been used to provoke equiaxed growth conditions. Mainly, these were adopted from the generally accepted theory of Hunt [8] and extensions for rapid solidification [9]. Numerical microstructural investigations on the mesoscale are normally based on existing nucleation models [10,11,12,13,14] that were developed for common casting processes using either cellular automata [10,11,12,13,14,15,16], Monte Carlo [17], or phase-field [18,19] methods. The most prominent models are from Rappaz [20] and Oldfield [21]. Besides the common columnar-to-equiaxed transition (CET) [5,19,22,23,24,25], other nucleation mechanisms have also been discovered. Here, new nuclei preferably occur at the melt-pool border during solidification [3,18,26,27,28,29,30,31]. However, there is still a lack of understanding. For instance, Helmer et al. [32] built samples with a columnar and equiaxed microstructure. On the basis of simulation analysis for a classical CET criterion, the columnar sample should have had a higher tendency towards a CET than that of the equiaxed sample. Thus, the classical theory is not suitable in this case to predict equiaxed microstructures. In general, the exact evolution of equiaxed grains during the layer-wise build during PBF is not examined well.

Here, we show current experiment and numerical results that offer insight on how, when, and where nucleation can occur during PBF except due to classical CET. For this purpose, single crystalline IN718 samples were built by selective electron beam melting (SEBM). The [100] direction of all single crystals was aligned along the build direction. In a second step, single melt lines were produced on top of the samples with varying process scan speed and beam power. On the basis of the experiments, the origin of nucleation was supported by results from our inhouse software SAMPLE${}^{2D}$. This can be used for extending the understanding of nucleation during PBF.

## 2. Experiments

### 2.1. Setup

**Figure 1:**
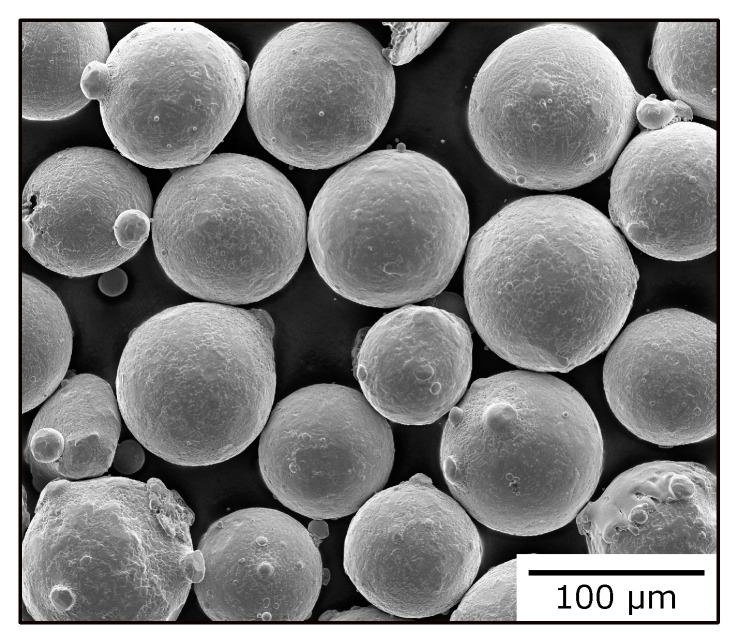
IN718 powder used in SEBM process. Image taken by Helios NanoLab DualBeam 600 FIB/SEM (FEI Company, Hillsboro, OR, USA).

Sixteen cuboid samples (15 × 15 × 20 mm^3^, XYZ) were built from IN718 powder on an Arcam A2 EBM machine (Arcam AB, Mölndal, Sweden), which was modified to meet the specifications of an Arcam A1. SEBM processing parameters are listed in Table 1. As a start plate, a polycrystalline IN718 disk with a thickness of 16 mm and a diameter of 136 mm was used. Samples were connected to the start plate via cylindrical support structures. Build temperature was held constant at 950 ${}^{\circ }C$, as measured by a thermocouple attached to the start plate. Build chamber pressure of 2 × 10^−3^ mbar under a helium atmosphere was applied during the build. As raw material, IN718 powder with a size distribution of 45 μm to 105 μm was used (TLS Technik GmbH & Co. Spezialpulver KG, Bitterfeld, Germany). The powder particles showed spherical morphology with few satellites and moderate residual porosity (compare Figure 1).

**Figure 2:**
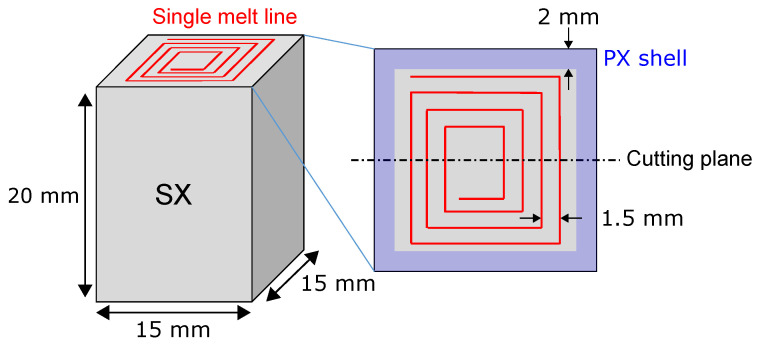
Single crystalline sample with spiral single melt line on top in addition to sample dimensions, polycrystalline shell, distance between adjacent melt lines, and cutting plane for metallographic preparation.

As shown in Reference [33], the process parameters led to single crystals (SX). The SX were surrounded by a polycrystalline (PX) shell with a thickness of about 2 mm perpendicular to the build direction. These single crystalline samples were used as a base for single-line experiments to investigate the predominant nucleation mechanism. Figure 2 schematically shows one sample with a spiral single melt line on top and the cutting plane for subsequent metallographic preparation. The distance between adjacent lines was 1.5 mm. The single crystals serve two purposes. First, melting single lines into the SEBM-prebuilt material were much closer to the actual SEBM process when compared to the more common way of melting into start plates, which are typically made by casting or forging. Second, a single crystalline base allows for a more clear-cut interpretation of nucleation phenomena. Due to the 2D nature of microsections used for analysis, grains piercing the observation plane could be incorrectly classified as instances of preceded nucleation when using a more typical columnar microstructure with different grain orientations. As there was only one main grain orientation with only a few small deviating grains in the present technical single crystal, this effect could be diminished.

A broad combination of beam powers *P* and scan velocities *v* was used as listed in Table 2. The parameter range was chosen in such a way that allowed for the generation of melt pools with sufficient melting depth while simultaneously suppressing evaporation phenomena due to excessive energy input. Before single-line melting, samples were heated to a build temperature of 950 ${}^{\circ }C$. For analysis, all samples were metallographically prepared as longitudinal microsections. They were subsequently etched with a V2A etching solution (mixture of HCl, HNO_3_, H_2_O, and Vogels special reagent) to reveal the dendritic microstructure and analyzed via light microscopy. For every parameter set, 7 lines were produced in 1 sample.

In order to gain further insight on grain orientation and nucleation, scanning-electron-microscopy (SEM) and electron-backscattered-diffraction (EBSD) measurements were performed on selected samples. All EBSD mappings were recorded with a step size of 750 nm. Measurements and postprocessing were performed on a Helios NanoLab DualBeam 600 FIB/SEM (FEI Company, Hillsboro, OR, USA) equipped with a NordlysNano EBSD detector using AZtecHKL and HKL CHANNEL5 software (Oxford Instruments, Abingdon, GB).

### 2.2. Nucleation and Crystal Growth

**Figure 3:**
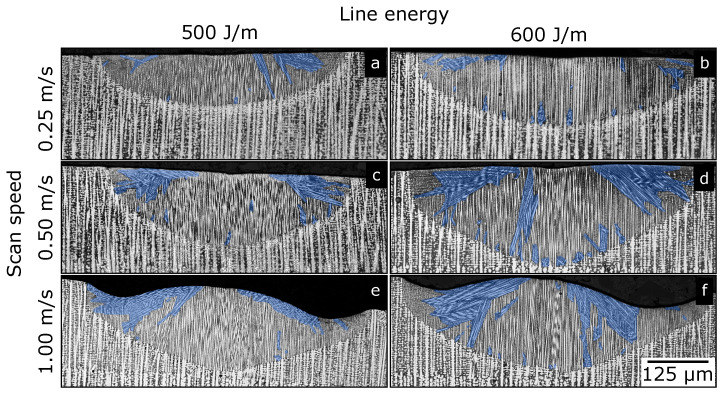
Exemplary microsections for different melting parameters (**a**–**f**) showing distinct evolution of new grains in single crystalline IN718 base material. For convenience, areas with new grains are highlighted in blue when their orientation was different from the base material. Proceeding grains from the base material are not marked.

Figure 3 shows six typical microsections from the experiments. For all 3 scan speeds, 2 different energy inputs are depicted. Shown samples are representative of all microsections. The base material was almost of a single crystalline nature with some deviations in each sample. As a result of cubic crystal symmetry, a transition of the growth direction from the single crystal aligned with the build direction to a 90${}^{\circ }$ growth mode was present at the flanks. This effect was also observed by Mokadem [28].

Every melt line showed pronounced areas with possible new grains (highlighted by blue in Figure 3). Nearly every new grain arose at the melt-pool border. At the melt-pool bottom, these grains did not grow far. At the flanks, the grains grew towards the top center of the melt pool. With an increasing melt pool, the fractional area of new grains increased.

**Figure 4:**
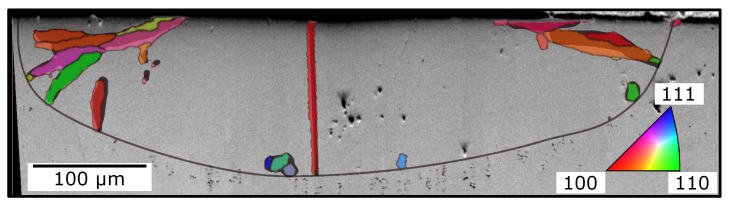
Melt pool of Sample 3 from Figure 3b with nucleation at melt-pool border shown as SEM image and electron-backscattered-diffraction (EBSD) overlay. For convenience, single crystal (SX) signal was deleted, and only new grains are shown in EBSD. In the underlying SEM image, new grains were manually highlighted for comparison with the EBSD signal. On the basis of the SEM image, the melt-pool border was inserted.

In Figure 4, Sample 3 from Figure 3b is shown as SEM image with a superimposed EBSD signal. Due to additional preparation before SEM/EBSD, the melt pool could not be directly compared to the corresponding microsection. Occasionally, new grains arose at the whole melt-pool border. SEM and EBSD images gave a good match. Only one new grain at the bottom right (light blue) was not observable in SEM. The EBSD image confirmed that the features found in microsections could be new grains nucleated at the melt-pool border. Badly oriented grains at the bottom were quickly overgrown. Only one grain in the middle had only slight deviation from the single crystal. Thus, it could survive.

**Figure 5:**
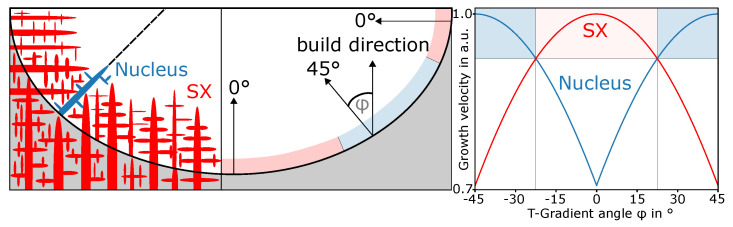
(**left**) Schematic melt pool with angle φ at border between build direction and temperature gradient. (**right**) Normalized growth velocities for same undercooling cos(α−|φ|) for different orientations α of SX base material (0${}^{\circ }$) and nucleus (45${}^{\circ }$) to the build direction. Areas where SX or nucleus grew faster because of better orientation marked either in red or blue in both subfigures.

To explain the growth behavior of new grains, Figure 5 schematically depicts local growth conditions along the border. This is based on the classical theory for growth competition by Walton and Chalmers [34]. The better that any of the main growth directions of a grain is aligned with the local temperature gradient, the faster it grows. In 2D space, a cubic crystal has four main growth directions with a 90${}^{\circ }$ rotational symmetry. Thus, the worst orientation of the temperature gradient is in the middle between two adjacent main growth directions. This leads to a maximal absolute deviation of 45${}^{\circ }$. The temperature gradient itself is always perpendicular to the melt-pool border.

On the right, the plot schematically shows the growth velocity of the SX base material grown in the build direction and a nucleus that is rotated by 45${}^{\circ }$. For the sake of simplicity, the curves follow a cosine function. Growth velocity was normalized with arbitrary maximal velocity. This leads to a maximal value of 1 when one of the main growth directions of a grain is aligned with the temperature gradient, and a minimal value of $\sqrt{2}/2$ for the highest deviation with an absolute value of 45${}^{\circ }$. One main growth direction of the SX (0${}^{\circ }$ towards the build direction) is aligned with the temperature gradient at the bottom. Additionally, two other main growth directions of the 0${}^{\circ }$ SX are aligned at the outermost melt-pool borders. At these locations, deviation between the main growth directions of the nucleus and the temperature gradient is maximal. At an angle for the temperature gradient of −45${}^{\circ }$ and 45${}^{\circ }$, the nucleus is aligned, and deviation for the SX is maximal. Because of rotational symmetry, it was sufficient to only consider the angle of the temperature gradient φ between −45${}^{\circ }$ and 45${}^{\circ }$. The areas for the fastest-growing crystals at a certain angle are marked in the top part of the right figure. The same areas for the angle are also depicted at the melt-pool border on the scheme on the left. In these areas, either the SX or the nucleus is better oriented towards the temperature gradient. Transitions between these areas are at −22.5${}^{\circ }$ and 22.5${}^{\circ }$. At these angles, deviation for SX and nucleus is equal. The theory correlates with the highlighted areas in Figure 3, where new grains at the bottom were overgrown by the SX and survived towards the flanks.

## 3. Simulation

### 3.1. Setup

To investigate the nucleation mechanism, a simulation with our inhouse software SAMPLE${}^{2D}$ [35] was performed. The software is based on a 2D lattice Boltzmann method that inherits melt-pool dynamics, melting/solidification, element-distribution variations, and evaporation. Here, solidification was not modeled because only the turning point between melting and the start of solidification was of interest. The parameter set from Sample 12 (P=400 W, v=1  m s^−1^) was chosen. Here, evaporation effects became visible in the sample (compare Figure 3e). The build temperature used in the experiments of 950 ${}^{\circ }C$ was initialized in the whole domain. For the 2D setup, all melt-pool movement in the beam direction was suppressed. To achieve mixing and homogenization in the 2D simulation, this specific parameter with evaporation was used. Additionally, some powder particles were applied to increase melt-pool movement in the 2D setup.

As a segregating element, only Nb was considered. The powder particles had a nominal Nb concentration of 5 wt-%. A Scheil-like microsegregation profile perpendicular to the beam direction was applied to the SX base material. Parallel to the beam, it was constant, imitating a prebuilt single crystal. On the basis of the primary dendrite arm spacing of the SX in experiments of about 13 μm, a rounded spacing for a Scheil distribution of 10 μm was chosen.

**Figure 6:**
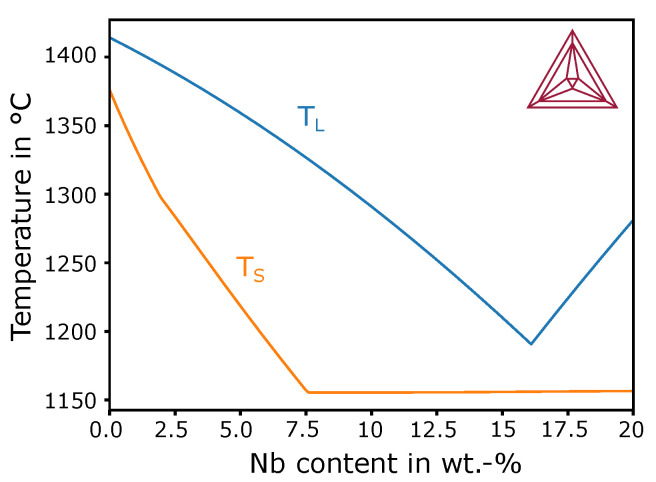
Quasibinary phase diagram of IN718-Nb calculated with ThermoCalc (2017b, TTNI8 database) up to 20 wt-% Nb content. Liquidus ($T_{L}$) and solidus ($T_{S}$) curves are shown.

To obtain sufficient resolution for this spacing, the cell size of the numerical grid was 500 nm. For numerical stability, the temporal increment needed to be set to 2 ns. Element diffusion was allowed in the melt, but ignored in the solid. The diffusion coefficient of Nb in the melt was calculated with the Einstein–Stokes–Sutherland equation [36,37]

(1) $$D=\frac{k_{b}T}{6\pi \eta r},$$

where Boltzmann constant, $k_{b}$; temperature, *T*; dynamic melt viscosity, η (see Table 3); and atomic radius of Nb, *r* ( 215 pm). Diffusion coefficient *D* was roughly set to 1 × 10^−9^ m^2^ s^−1^ for temperature around the liquidus temperature of IN718 of 1359 ${}^{\circ }C$ (see Figure 6 at 5 wt-%). Liquidus and solidus temperature levels were locally evaluated on the basis of the current composition (Nb content) based on a quasibinary phase diagram (IN718-Nb) calculated with ThermoCalc (see Figure 6). The used material parameters are summarized in Table 3. Temperature-dependent thermal conductivity was approximated by a linear fit in the solid and liquid phases on the basis of values given in Table 3.

### 3.2. Remelting Simulation

**Figure 7:**
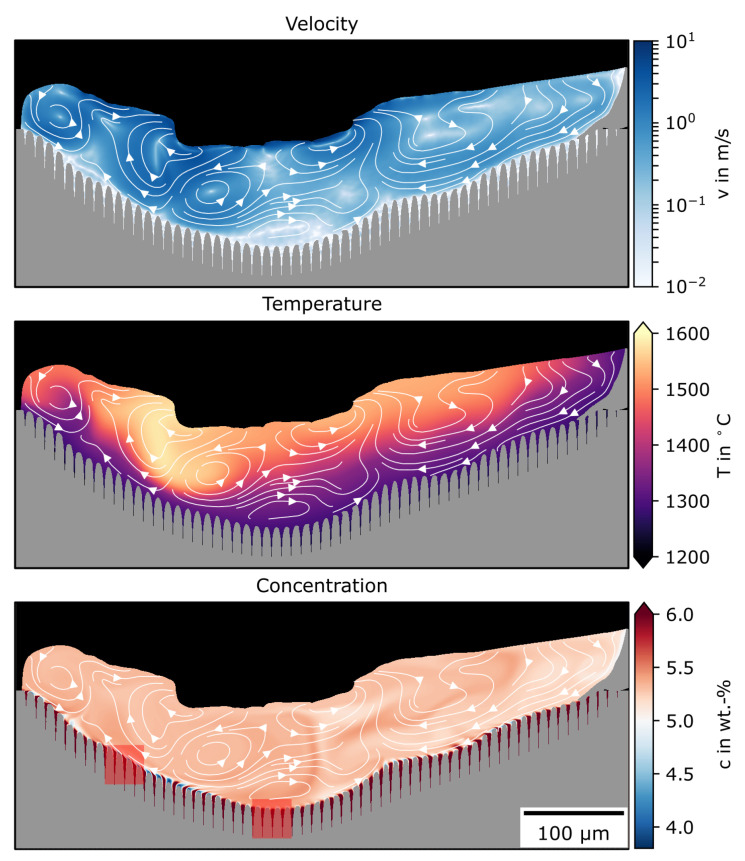
Simulation results of velocity, temperature, and concentration distribution when maximal melt-pool depth was reached during remelting of predefined periodical Nb composition. Stream lines show current velocity field. For the concentration field, two areas are marked for further investigations.

Figure 7 shows a snapshot from the simulation when the maximal melt-pool depth was reached during the remelting of the predefined Nb distribution. The velocity, temperature, and Nb concentration with stream lines of the velocity field are depicted. Due to capillarity, wetting, and evaporation, the melt pool was highly dynamic, with velocities up to 10 m s^−1^. This caused inhomogeneous temperature distribution with an uneven melt-pool border.

Furthermore, the high convective flow resulted in the nearly complete mixing of the predefined concentration profile. Concentration was around the nominal concentration of Nb in IN718 of 5 wt-% nearly everywhere in the melt pool. Additionally, the melt border followed the shape of the former dendrite structure due to segregation remelting.

**Figure 8:**
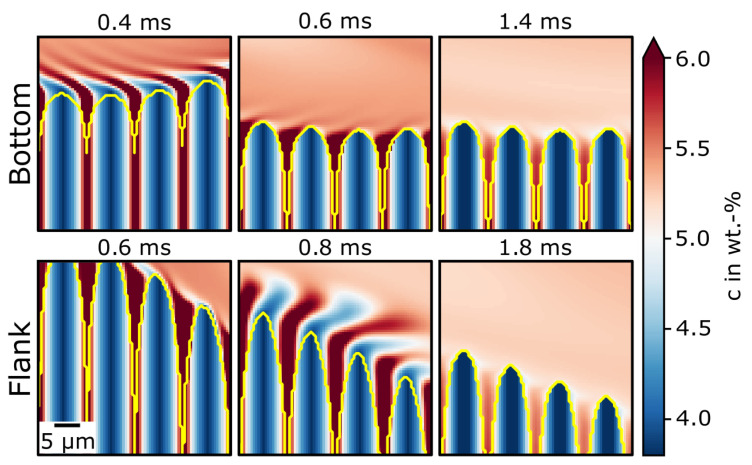
Temporal evolution of Nb composition during remelting at bottom and flanks (comparing Figure 7) at different times. Solid–liquid interface highlighted in yellow.

The importance of convective transport not for remelting but for solidification was shown and emphasized by Zhao et al. [41]. They found that solidification velocities are strongly influenced by melt flow. Figure 8 shows the temporal evolution of the concentration distribution at the melt-pool bottom and flank for the areas marked in Figure 7. The last time step depicts the turning point between melting and solidification for each area. In both cases, a similar effect was apparent. During melting, the concentration homogenized above the melt-pool border. Between the former dendrites, homogenization was much slower.

In the majority of the melt, the concentration was mixed by the convective flow. In the confined space between the dendrites, the concentration could only be homogenized by diffusion. Thus, the concentration in the interdendritic region was higher than that in the rest of the melt pool. Furthermore, at the transition between interdendritic region and melt pool, the concentration was very close to 5 wt-%. In the melt pool itself, it was slightly higher in the shown sections (comparing the last time step for bottom and flank in Figure 8). This led to a small concentration sink at the transition area with an increased concentration gradient.

**Figure 9:**
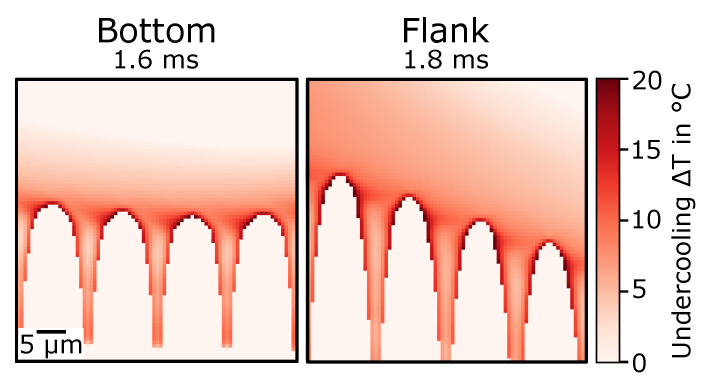
Undercooling after remelting at two locations in melt pool (compare Figure 7) at (**left**) melt-pool bottom at 1.6 ms and at (**right**) flanks at 1.8 ms.

Figure 9 shows the undercooling at the turning point from melting to solidification. In the simulation, no solidification was considered. Thus, the turning point was rather arbitrary, and the time when the undercooling was plotted was shortly after undercooling became apparent. The different times for the bottom and flank were caused by different times when the undercooling started to locally evolve. Undercooling was calculated on the basis of local liquidus temperature and local temperature. The undercooling was highest at the dendrite tips. It gradually decreased to zero above the dendrites. In the transition areas from interdendritic region to melt pool, undercooling was also high because of the concentration gradient. Deeper in the interdendritic space, undercooling decreased.

## 4. Cooling-Rate Determination

### 4.1. Setup

To investigate the influence of different energy inputs on cooling rate, further single-line simulations were conducted. Scan speed and the line energy were varied following the parameters defined in Table 2. Cell size and temporal increment were increased to 5 μm and 200 ns to decrease calculation time. Furthermore, evaporation was neglected, and no powder was applied. Nb content was set to 5 wt-% in the whole domain. To estimate cooling rate, the corresponding liquidus isotherm was tracked. Lastly, the mean for all cells at the melt-pool border was calculated for every simulation.

### 4.2. Influence of Line Energy on Cooling Rate

**Figure 10:**
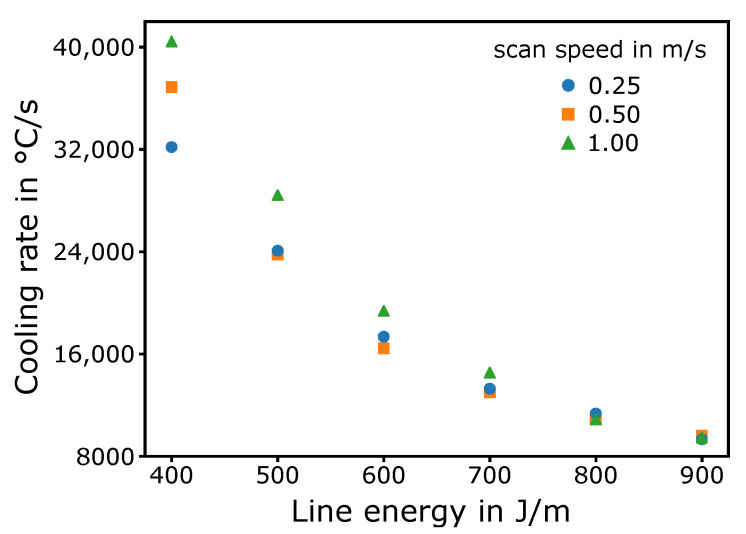
Cooling rate at melt-pool border for several scan speeds and line energies.

In Figure 10, cooling rate at the melt-pool border is depicted for several line energies and scan speeds obtained from the single-line simulations. For the lowest line energy of 400 J m^−1^, the highest cooling rate of around 40,000 ${}^{\circ }$C s${}^{-1}$ appeared for the highest scan speed of 1 m s^−1^. With decreasing scan speed, the cooling rate also decreased for 400 J m^−1^. For increasing line energies, cooling rate decreased steadily; for 900 J m^−1^, it was around 9000 °C s^−1^ for all scan speeds.

## 5. Discussion

Experiments showed that melt pools show preferably nucleation at the melt-pool border. After nucleation, the survival of grains follows a regular grain-selection process, where better-oriented grains overgrow others. Thus, for the SX base material, only new grains at the flanks survived.

For nucleation, there are two prerequisites. First, a nucleation site for heterogeneous nucleation (homogeneous nucleation is irrelevant for practical use). Second, undercooling as driving force.

In order to produce new nucleation sites, dendrite fragmentation is an often discussed theory controversy [42,43,44,45,46,47,48,49]. Drezet et al. [30] claimed that high velocities in a melt pool lead to dendrite fragmentation, leading to new grains at the melt-pool border. However, due to very low dendrite arm spacings and poor formation of secondary arms, we doubt that fragmentation in the interdendritic space caused by the constitutional remelting of secondary arms is present. It is more realistic that high melting oxides, nitrides, or carbides in the interdendritic region during remelting [50,51] can act as nucleation sites. For example, aluminum oxide has a melting point far above 2000 ${}^{\circ }C$ [52]. Temperature at the bottom is only slightly above the local liquidus temperature of IN718 of around 1359 ${}^{\circ }C$ (compare Figure 6).

The second prerequisite was examined by simulation. Classically, undercooling increases close to the solidification front by constitutional undercooling leading to nucleation events towards the end of solidification (CET). Zhang et al. [53] proposed that altering constitutional undercooling by changing the composition can lead to a complete CET right at the beginning of solidification. However, this cannot explain the fact that new grains can only be found at the beginning and not afterwards as constitutional undercooling increases during solidification. Furthermore, Liu et al. [31] reported that the temperature gradient is responsible for nucleation at the melt-pool border. However, no further explanation was given. These approaches are not suitable to explain nucleation exclusively at the melt-pool border. Our simulation showed that remelting segregations led to recreation of the shape of the dendritic structure at the border. Additionally, mixing in the melt pool effectively homogenized these remelted segregations except for in the interdendritic regions at the border. This led to increased undercooling in this area right at the beginning of solidification. Thus, a condition basically close to classical constitutional undercooling was created right at the turning point. Furthermore, close to the interdendritic space, new nuclei could grow freely until the actual front arrived. Thus, they had time to reach a critical size to survive.

**Figure 11:**
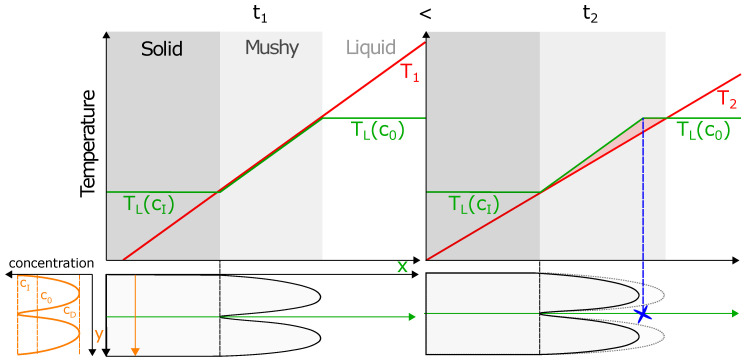
Schematic representation of liquidus temperature $T_{L}$ and current temperatures ($T_{1}$ and $T_{2}$) at turning point from melting to solidification (time $t_{1}$) and shortly after solidification started (time $t_{2}$). Due to a concentration gradient in the mushy zone, a constitutional undercooled region occurred where nucleation could happen. Former concentration distribution in solid with nominal concentration $c_{0}$, maximal concentration in former interdendritic regions $c_{I}$, and minimal concentration in former dendrite cores $c_{D}$ are shown.

A summary for the possible mechanism behind the border nucleation is schematically shown in Figure 11.

Two times, $t_{1}$ and $t_{2}$, are depicted. The first shows a situation right at the turning point between melting and solidification. The second shows the situation right after solidification started. Current and local liquidus temperatures are illustrated along the interdendritic space. In the solid zone, high Nb concentrations lead to a low liquidus temperature. In the mixed melt pool with the nominal Nb concentration, liquidus temperature is high. For simplicity, the two levels are linearly connected. This is only a schematic approximation. Temperature is linearly approximated in a way that, at the turning point, the temperature in the mushy zone is equal to the liquidus temperature at $t_{1}$. At time $t_{2}$, temperature gradient shrinks due to cooling. The concentration field stays constant for convenience. This is justified by the transient melt pool (compare Figure 8 and Figure 9).

A decrease in temperature causes the dendrite tips to grow a short distance. Additionally, it results in a constitutional undercooled zone in the interdendritic space between two tips. Nucleation can happen in this undercooled regions. However, here, the solidification front is static at the transition point. Thus, compared to a moving front from a classical view point, new nuclei have more time to grow. This increases the probability for them to survive at the melt-pool border. Lastly, this can lead to the nucleation phenomenon seen in the experiments. After nucleation, the survival rate of new grains depends only on growth competition, as depicted in Figure 5.

At last, Figure 10 shows how different energy inputs can influence the mechanism. By increasing the energy input, the cooling rate at the melt-pool border decreases. Accordingly, the time for diffusion at the border increases. Therefore, the transient concentration gradient in the interdendritic region shrinks, leading to a decrease in constitutional undercooling produced by remelting. This can explain why single crystals can be produced by PBF processes [33,54,55]. With common hatching methods, the cooling rate can be further decreased, and nucleation at the border vanishes.

## 6. Summary and Conclusions

In this paper, the nucleation mechanism during PBF was studied in detail for the first time. Nucleation primarily happened at the melt-pool border of individual melt lines at the onset of solidification, but not afterwards. The entirety of the melt-pool borders was cluttered with new nuclei. Nevertheless, only nuclei that were better aligned with the local temperature gradient could survive. This normally happens at an angle towards the built direction of around 45${}^{\circ }$ for cubic crystals. The current hypothesis is that the underlying mechanism is based on constitutional undercooling obtained by remelting nonuniform concentration distributions caused by microsegregation. During the melting of the segregations, the former shape of the dendrite structure was revealed.

On the basis of our simulation results, homogenization happened above the former dendrites, but not between them. This produced a transient concentration gradient, leading to constitutional undercooling in the interdendritic space right at the onset of solidification. This phenomenon could be the origin of the melt-pool-border nucleation. For producing equiaxed structures, this nucleation mechanism could be exploited. However, this needs the precise adjustment of process parameters. Increased energy inputs could decrease constitutional undercooling produced by remelting.

For more quantitative investigations of the mechanism, additional simulations and experiments are necessary. These results can be used to further adjust nucleation models used for layered manufacturing techniques under nonequilibrium solidification conditions to predict process parameters for desired microstructures.

Lastly, this can be used to select different process parameters within one part to produce different microstructures in different locations. For example, the root of a turbine blade could be built with equiaxed microstructures to increase dynamic strength, whereas the airfoil could be produced with a single crystalline microstructure to improve creep resistance.

## Figures and Tables

**Table 1:** Selective electron beam melting (SEBM) processing parameters for single crystalline base samples.

Beam Power	Scan Speed	Line Offset	Layer Thickness
546 W	7 m/s	30 μm	50 μm

**Table 2:** Beam parameters for single-line experiments. Line-energy stepping was 100 J m^−1^.

Samples	Line Energy (P/v)	Beam Power *P*	Scan Speed *v*
1–6	400–900 J m^−1^	100–225 W	0.25 m s^−1^
7–11	400–800 J m^−1^	200–400 W	0.50 m s^−1^
12–16	400–800 J m^−1^	400–800 W	1.00 m s^−1^

**Table 3:** Material parameters for simulations [38,39,40].

Parameter	Value	Unit
Density	7580	kg m^−3^
Viscosity	4.9 × 10${}^{-3}$	Pas
Atomic weight	56.6	
Atomic number	26.73	
Surface tension	1.73	J m^−2^
boiling temperature	2917	${}^{\circ }C$
heat capacity of solid	652	J kg^−1^
heat capacity of liquid	778	J kg^−1^
Thermal conductivity (solid) at 1000 K	22.4	W m^−1^ K^−1^
Thermal conductivity (solid) at 1500 K	30.7	W m^−1^ K^−1^
Thermal conductivity (liquid) at 1700 K	28.1	W m^−1^ K^−1^
Thermal conductivity (liquid) at 2100 K	33.5	W m^−1^ K^−1^
Heat of fusion	2.27 × 10${}^{5}$	J kg^−1^
Heat of vaporization	6.98 × 10${}^{6}$	J kg^−1^
Diffusion coefficient of Nb in Ni	1.0 × 10${}^{-9}$	m^2^ s^−1^

## References

[1] Popovich A., Sufiiarov V., Borisov E., Polozov I., Masaylo D. (2018). Design and manufacturing of tailored microstructure with selective laser melting. Mater. Phys. Mech..

[2] Koepf J.A., Gotterbarm M.R., Markl M., Körner C. (2018). 3D multi-layer grain structure simulation of powder bed fusion additive manufacturing. Acta Mater..

[3] Helmer H. (2017). Additive Fertigung Durch Selektives Elektronenstrahlschmelzen der Nickelbasis Superlegierung IN718: Prozessfenster, Mikrostruktur und Mechanische Eigenschaften. Ph.D. Thesis.

[4] Kirka M.M., Lee Y., Greeley D.A., Okello A., Goin M.J., Pearce M.T., Dehoff R.R. (2017). Strategy for Texture Management in Metals Additive Manufacturing. JOM.

[5] Raghavan N., Dehoff R., Pannala S., Simunovic S., Kirka M., Turner J., Carlson N., Babu S.S. (2016). Numerical modeling of heat-transfer and the influence of process parameters on tailoring the grain morphology of IN718 in electron beam additive manufacturing. Acta Mater..

[6] Raghavan N., Simunovic S., Dehoff R., Plotkowski A., Turner J., Kirka M., Babu S. (2017). Localized melt-scan strategy for site specific control of grain size and primary dendrite arm spacing in electron beam additive manufacturing. Acta Mater..

[7] Lee Y., Kirka M., Dinwiddie R., Raghavan N., Turner J., Dehoff R., Babu S. (2018). Role of scan strategies on thermal gradient and solidification rate in electron beam powder bed fusion. Addit. Manuf..

[8] Hunt J. (1984). Steady state columnar and equiaxed growth of dendrites and eutectic. Mater. Sci. Eng..

[9] Gäumann M., Trivedi R., Kurz W. (1997). Nucleation ahead of the advancing interface in directional solidification. Mater. Sci. Eng. A.

[10] Akram J., Chalavadi P., Pal D., Stucker B. (2018). Understanding grain evolution in additive manufacturing through modeling. Addit. Manuf..

[11] Li X., Tan W. (2018). Numerical investigation of effects of nucleation mechanisms on grain structure in metal additive manufacturing. Comput. Mater. Sci..

[12] Panwisawas C., Qiu C., Anderson M.J., Sovani Y., Turner R.P., Attallah M.M., Brooks J.W., Basoalto H.C. (2017). Mesoscale modelling of selective laser melting: Thermal fluid dynamics and microstructural evolution. Comput. Mater. Sci..

[13] Nath P., Hu Z., Mahadevan S. Modeling and Uncertainty Quantification of Material Properties in Additive Manufacturing. Proceedings of the 2018 AIAA Non-Deterministic Approaches Conference.

[14] Herriott C., Li X., Kouraytem N., Tari V., Tan W., Anglin B., Rollett A.D., Spear A.D. (2019). A multi-scale, multi-physics modeling framework to predict spatial variation of properties in additive-manufactured metals. Model. Simul. Mater. Sci. Eng..

[15] Li X., Tan W. 3-Dimesional Cellular Automata Simulation of Grain Structure in Metal Additive Manufacturing Process. Proceedings of the 28th Annual International Solid Freeform Fabrication Symposium—An Additive Manufacturing Conference.

[16] Lopez-Botello O., Martinez-Hernandez U., Ramírez J., Pinna C., Mumtaz K. (2017). Two-dimensional simulation of grain structure growth within selective laser melted AA-2024. Mater. Des..

[17] Rodgers T.M., Madison J.D., Tikare V. (2017). Simulation of metal additive manufacturing microstructures using kinetic Monte Carlo. Comput. Mater. Sci..

[18] Wang X., Liu P.W., Ji Y., Liu Y., Horstemeyer M.H., Chen L. (2019). Investigation on Microsegregation of IN718 Alloy During Additive Manufacturing via Integrated Phase-Field and Finite-Element Modeling. J. Mater. Eng. Perform..

[19] Shimono Y., Oba M., Nomoto S., Koizumi Y., Akihiko C. Numerical Investigation of Solidification in Additive Manufacturing of Ti Alloy by Multi-Phase Field Method. Proceedings of the 28th Annual International Solid Freeform Fabrication Symposium—An Additive Manufacturing Conference, SFF 2017.

[20] Rappaz M. (1989). Modelling of microstructure formation in solidification processes. Int. Mater. Rev..

[21] Oldfield W. (1966). Quantitative approach of solidification casting—Freezing of cast iron. Trans. ASM.

[22] Yan F., Xiong W., Faierson E.J. (2017). Grain Structure Control of Additively Manufactured Metallic Materials. Materials.

[23] Gäumann M., Henry S., Cléton F., Wagnière J.D., Kurz W. (1999). Epitaxial laser metal forming: Analysis of microstructure formation. Mater. Sci. Eng. A.

[24] Gäumann M., Bezençon C., Canalis P., Kurz W. (2001). Single-crystal laser deposition of superalloys: Processing– microstructure maps. Acta Mater..

[25] Gäumann M., Kurz W. (1998). Why Is It so Difficult to Produce an Equiaxed Microstructure during Welding?.

[26] Karimi P., Sadeghi E., Ålgårdh J., Andersson J. (2019). EBM-manufactured single tracks of Alloy 718: Influence of energy input and focus offset on geometrical and microstructural characteristics. Mater. Charact..

[27] Dezfoli A., Ansari R., Hwang W.S., Huang W.C., Tsai T.W. (2019). Determination and controlling of grain structure of metals after laser incidence: Theoretical approach. Sci. Rep..

[28] Mokadem S. (2004). Epitaxial Laser Treatment of Single Crystal Nickel-Base Superalloys. Ph.D. Thesis.

[29] Thijs L., Kempen K., Kruth J.P., Humbeeck J.V. (2013). Fine-structured aluminium products with controllable texture by selective laser melting of pre-alloyed AlSi10Mg powder. Acta Mater..

[30] Drezet J.M., Mokadem S. (2006). Marangoni Convection and Fragmentation in LASER Treatment. Mater. Sci. Forum.

[31] Liu X., Zhao C., Zhou X., Shen Z., Liu W. (2019). Microstructure of selective laser melted AlSi10Mg alloy. Mater. Des..

[32] Helmer H., Bauereiß A., Singer R., Körner C. (2016). Grain structure evolution in Inconel 718 during selective electron beam melting. Mater. Sci. Eng. A.

[33] Gotterbarm M.R., Rausch A.M., Körner C. (2020). Fabrication of Single Crystals through a µ-Helix Grain Selection Process during Electron Beam Metal Additive Manufacturing. Metals.

[34] Walton D., Chalmers B. (1959). The origin of the preferred orientation in the columnar zone of ingots. Trans. Metall. Soc. AIME.

[35] Markl M., Rausch A.M., Küng V.E., Körner C. (2019). SAMPLE: A Software Suite to Predict Consolidation and Microstructure for Powder Bed Fusion Additive Manufacturing. Adv. Eng. Mater..

[36] Einstein A. (1905). Über die von der molekularkinetischen Theorie der Wärme geforderte Bewegung von in ruhenden Flüssigkeiten suspendierten Teilchen. Ann. Phys..

[37] Sutherland W. (1905). Dynamical theory of diffusion for non-electrolytes and the molecular mass of albumin. Phil. Mag..

[38] Rai A., Markl M., Körner C. (2016). A coupled Cellular Automaton–Lattice Boltzmann model for grain structure simulation during additive manufacturing. Comput. Mater. Sci..

[39] Sames W.J., Unocic K.A., Dehoff R.R., Lolla T., Babu S.S. (2014). Thermal effects on microstructural heterogeneity of Inconel 718 materials fabricated by electron beam melting. J. Mater. Res..

[40] Pottlacher G., Hosaeus H., Kaschnitz E., Seifter A. (2002). Thermophysical properties of solid and liquidInconel 718 Alloy. Scand. J. Metall..

[41] Zhao Y., Koizumi Y., Aoyagi K., Wei D., Yamanaka K., Chiba A. (2019). Molten Pool Behavior and Effect of Fluid Flow on Solidification Conditions in Selective Electron Beam Melting (SEBM) of a Biomedical Co-Cr-Mo Alloy. Addit. Manuf..

[42] O’Hara S., Tiller W. (1967). On the mechanisms of crystal multiplication during solidification in the presence of fluid motion. Trans. Metall. Soc. AIME.

[43] Mathiesen R.H., Arnberg L., Bleuet P., Somogyi A. (2006). Crystal fragmentation and columnar-to-equiaxed transitions in Al-Cu studied by synchrotron X-ray video microscopy. Metall. Mater. Trans. A.

[44] Kou S. (2003). Weld Metal Solidification I: Grain Structure. Welding Metallurgy.

[45] Hellawell A., Liu S., Lu S.Z. (1997). Dendrite fragmentation and the effects of fluid flow in castings. JOM.

[46] Herlach D., Eckler K., Karma A., Schwarz M. (2001). Grain refinement through fragmentation of dendrites in undercooled melts. Mater. Sci. Eng. A.

[47] Liu S., Lu S.Z., Hellawell A. (2002). Dendritic array growth in the systems NH_4_Cl-H_2_O and [CH_2_CN]_2_-H_2_O: The detachment of dendrite side arms induced by deceleration. J. Cryst. Growth.

[48] Beckermann C. (1997). Modeling segregation and grain structure development in equiaxed solidification with convection. JOM.

[49] Ruvalcaba D., Mathiesen R., Eskin D., Arnberg L., Katgerman L. (2007). In situ observations of dendritic fragmentation due to local solute-enrichment during directional solidification of an aluminum alloy. Acta Mater..

[50] Matz J., Eagar T.W. (2002). Carbide formation in alloy 718 during electron-beam solid freeform fabrication. Met. Mater. Trans. A.

[51] Yu H., Hayashi S., Kakehi K., Kuo Y.L. (2019). Study of Formed Oxides in IN718 Alloy during the Fabrication by Selective Laser Melting and Electron Beam Melting. Metals.

[52] Patnaik P. (2003). Handbook of Inorganic Chemicals.

[53] Zhang D., Qiu D., Gibson M.A., Zheng Y., Fraser H.L., StJohn D.H., Easton M.A. (2019). Additive manufacturing of ultrafine-grained high-strength titanium alloys. Nature.

[54] Körner C., Ramsperger M., Meid C., Bürger D., Wollgramm P., Bartsch M., Eggeler G. (2018). Microstructure and Mechanical Properties of CMSX-4 Single Crystals Prepared by Additive Manufacturing. Metall. Mater. Trans. A.

[55] Chauvet E., Tassin C., Blandin J.J., Dendievel R., Martin G. (2018). Producing Ni-base superalloys single crystal by selective electron beam melting. Scr. Mater..

